# Supplementary material for: A Dataset of Social-Psychological and Emotional Reactions During the COVID-19 Pandemic Across Four European Countries
Source: J Open Psychol Data. 2023 Jul 11;11:11. doi: 10.5334/jopd.86 (PMC12273686; doi:10.5334/jopd.86)
Supplement: Qualtrics Codebook. — A Dataset of Social-Psychological and Emotional Reactions during the COVID-19 Pandemic across Four European Countries.pdf. [file jopd-11-86-s1.pdf]

Qualtrics Codebook

Index

Variable Names in the CSV dataset file, plus the additional prefix *A*, refer to the numbering in the codebook. For example, A3.1 refers to item 3.1 in the codebook.

Variable Labels indicate the readable description of each variable and are only included in the codebook. For example, *Gender* indicates item 3.1.

Value Labels indicate the labels for each value chosen by the respondents. For example, for *Gender* the labels were as follows (1=male, 2=female, 3=other).

Reversed Items are indicated with an asterisk * in the codebook and CSV dataset file.

Intro Items (2.1, 4.1, 8.1, 19.1, 26.1, 28.1, 30.1, 33.1, 35.1, 37.1, 39.1) are meant to introduce respondents to a series of questions or a specific topic, in addition to the informed consent (1.1).

Attention-Check Item 18.1 indicated an attention-check and does not require any coding.

Country The countries of the collected data are indicated here (1=Germany, 2=Spain, 3= Netherlands, 4= United Kingdom).

RespondentID Consecutive numbers were assigned to all valid respondents (N=2031).

Informed Consent

1.1. Dear participant, thank you for taking part in our survey. Before you begin, please make sure you read the following information:

Purpose of the investigation
This survey is about the effects of the Coronavirus on your attitudes, feelings and behavior. The questionnaire presented below investigates how you think about yourself, your country, your prospects, and your government. There are no right or wrong answers. This is about your ideas, thoughts and feelings. In total, this questionnaire will take about 15 minutes to complete.

Voluntary participation
There are no consequences if you decide not to participate in this study. You may withdraw your consent to allow the researchers to use your answers and data within seven days following your participation. If you choose to withdraw from the study, your data will be deleted permanently.

Your privacy is guaranteed
Your personal information (about who you are) is entirely confidential and will not be shared without your explicit consent. The data will be anonymized to make sure that no one can trace your data back to you personally (following the EU’s General Data Protection Regulation GDPR). Only data which has been completely anonymized may be shared among researchers.

I consent to the following:
- I am 18 year or older.
- I have read and understood the information above.
- I consent to participate in this study.
- I consent to allow the researchers of this study to use my anonymized data, and to share them with other researchers interested in the topic.
- I understand that I can withdraw this consent at any time.

- Yes, I hereby give consent for my completely anonymized data to be used for the purposes of this study and shared with other researchers.
- No, I do not give consent and I wish to stop with participating in this study.

Demographics Intro

2.1. First, we would like to ask you some background questions:

Demographics

3.1. Gender (1=male, 2=female, 3=other)

3.2. Age (1 = 18 – 24, 2 = 25 – 34, 3 = 35 – 44, 4 = 45 – 54, 5 = 55 – 64, 6 = 65 – 74, 7 = 75 – 84, 8 = 85 or older)

3.3. Country of birth (based on ISO 3166 country codes)

3.4. Country of first citizenship (passport) (based on ISO 3166 country codes)

3.5. Marital status (1=single, 2=in a relationship, 3=married, 4=divorced, 5=widowed)

3.6. Employment status (1=employed, 2=self-employed, 3=unemployed, 4=student, 5=retired, 6=inapplicable)

Subjective Social Status (1 = lowest, 10 = highest)
3.7. Imagine a ladder with 10 rungs - where do you see yourself standing in terms of your socio-economic status in your society?

3.8. Religion (1=Protestant, 2=Roman-Catholic, 3=Muslim, 4=Jewish, 5=Russian-Orthodox, 6=Greek-Orthodox, 7=Hindu, 8=Buddhist, 9=Agnostic, 10=Atheist, 11=Spiritual, 12=Non-Religious

Political Orientation (-5 = Left-Wing, 0 = Centrist, 5 = Right-Wing)
3.9. Where would you place your political orientation on this spectrum?

Left-Wing Centrist Right-Wing
-5 -4 -3 -2 -1 0 1 2 3 4 5

Education
3.10. What is your highest education level completed?

(1=Less than high school degree, 2=High school degree or equivalent, 3=Some college but no degree, 4=Vocational/technical degree, 5=Bachelor's degree, 6=Master's degree, 7=Professional doctorate (MD, JD), 8=Doctoral degree (PhD))

Anxiety about Coronavirus Intro
4.1. We examine the effects of the coronavirus crisis on your attitudes, thinking and behavior. To start with, we want to ask you some questions about your feelings and thoughts.

(1 =Not at all, 10 = Extremely)

Anxiety about Coronavirus
5.1. I am concerned about the effects of the coronavirus.
5.2. I am worried that my family may be affected by the coronavirus.
*5.3. I think people are exaggerating the contagious effects of the coronavirus.
5.4. I think about the coronavirus all the time.
5.5. I feel disgusted with sick people infected with the coronavirus.

Feeling Status (1 = Negative, 10 = Positive)
6.1. How do you feel right now?

Coronavirus Infection Status (Self and Friends)
7.1. Are you or have you been infected with the coronavirus?
7.2. Do you know people in your immediate social environment who are or have been infected with the coronavirus?

1 = I Don't know
2 = No
3 = Yes, but not confirmed yet
4 = Yes, confirmed

Threats Intro
8.1. Next, we would like to ask you some questions about other concerns that you may have.

Threats (Realistic/Symbolic) (1=Not at all, 2=Low, 3=Slightly, 4=Neutral, 5=Moderately, 6=Very, 7=Extremely)

9.1. I am afraid that I will lose my job in the near future.
9.2. I fear that it will be very difficult for me to find proper housing.
*9.3. I expect that my living standards will improve in the coming years.
9.4. I am anxious about what the future will bring.
9.5. The norms and values that I find important are no longer important in [country].
9.6. I think women should stay home and take care of the children.
9.7. The immigration of people from many foreign countries is a threat to my values.
*9.8. I think that [country] traditions will benefit from people of multicultural influences.

Selection of News Headlines
10.1. We are interested in the type of news that people like to read. We will present you with two different newspaper headlines and ask you which one you would prefer to read. There are no wrong or right answers, we are just interested in your preferences.

News Headlines (1. Coronavirus vs. Non-Threatening)
11.1. Please choose which of the following headlines you would prefer to read.
The disastrous effects of the coronavirus crisis on the worldwide economy.
The role of coral reefs in protecting sea life.

11.2. Please choose which of the following headlines you would prefer to read.
The top-10 tourist attractions in the world.
The coronavirus is the deadliest virus known in the history of humankind.

11.3. Please choose which of the following headlines you would prefer to read.
Why it is so difficult to combat the coronavirus?
How to develop better social skills?

11.4. Please choose which of the following headlines you would prefer to read.
How do social media affect youth reading?
Is reinfection by the coronavirus a possibility?

News Headlines (2. Threatening vs. Non-Threatening)
12.1. Please choose which of the following headlines you would prefer to read.
The earth is warming up much more rapidly than we predicted.
Eight ways to boost your presentation.

12.2. Please choose which of the following headlines you would prefer to read.
How can you make online meetings the most effective?
Why is it so difficult to solve the refugee crisis?

12.3. Please choose which of the following headlines you would prefer to read.
The role of social media in the music industry.
Why should Europe close its borders?

12.4. Please choose which of the following headlines you would prefer to read.
Wind turbines are not sufficient in reducing global warming.
How drones can help us in many ways.

News Headlines (3. Coronavirus vs. Threatening)
13.1. Please choose which of the following headlines you would prefer to read.
How much does immigration cost us?
Is it impossible to control the spread of the coronavirus?

13.2. Please choose which of the following headlines you would prefer to read.
The majority of the world population will be infected by the coronavirus.
Many countries in Europe suffer from financial corruption.

13.3. Please choose which of the following headlines you would prefer to read.
Can the coronavirus be eliminated?
How can we stop the polar circles from melting so rapidly?

13.4. Please choose which of the following headlines you would prefer to read.
Alert of a terrorist attack somewhere in Europe.

The mutations of the coronavirus continue to increase.

News Headlines (4. Conspiracy vs. Non-Conspiracy)
14.1. Please choose which of the following headlines you would prefer to read.
The coronavirus, a virus created to change the present world order.
Facts and figures about the spread of the coronavirus.

14.2. Please choose which of the following headlines you would prefer to read.
Who is benefiting from the coronavirus crisis and why?
The economic consequences of the coronavirus crisis.

14.3. Please choose which of the following headlines you would prefer to read.
A deadly virus that hits the elderly and the weak: Hidden benefits of the coronavirus.
How the coronavirus mutates: facts and prospects.

14.4. Please choose which of the following headlines you would prefer to read.
Scientific milestones regarding the coronavirus.
The coronavirus is a biological weapon and not a simple virus.

Threat Estimation (1 = extremely unlikely, 10 = extremely likely)
15.1. Next, please indicate your most likely estimates in the sliders below about things happening to you or in the world around us.

Threat Estimation (Coronavirus)
16.1. How likely do you think it is that you will contract the coronavirus yourself?
16.2. How likely do you think it is that someone close to you dies because of the coronavirus?
*16.3. How likely do you think it is that a therapy for the coronavirus is found soon?
*16.4. How likely do you think it is that a vaccine against the coronavirus will be developed?
16.5. How likely do you think it is that more than 10.000 people in your country will die due to the coronavirus?
16.6. How likely do you think it is that the economic crisis due to the coronavirus will affect you?

Threat Estimation (Climate, Symbolic Material and Safety)
17.1. How likely do you think it is that we cannot feed the world's population anymore by 2050?
17.2. How likely do you think it is that there will be food scarcity in your country?
17.3. How likely do you think it is that you will witness at least one natural catastrophe in your lifetime?
17.4. How likely do you think it is that your country will be severely affected by sea levels rising?
17.5. How likely do you think it is that your bank will be confronted with a severe cyber-attack?
17.6. How likely do you think it is that there will be international wars for clean water in your lifetime?
17.7. How likely do you think it is that traditions of your country will disappear due to the increase of immigrants and asylum seekers?
17.8. How likely do you think it is that refugees will damage the language in your country?

17.9. How likely do you think it is that foreigners will ‘foul’ the values of your country?
*17.10. How likely do you think it is that practices in your country can benefit from foreign cultures?
17.11. How likely do you think it is that your social relationships will be severely affected in the coming months?
17.12. How likely do you think it is that there will be more political polarization in your country?

Attention-Check
18.1. Based on the instruction below, what would you say your favorite city in Europe is? This is a simple question - when asked for your favorite city, you need to select Lisbon.

1=London
2=Berlin
3=Madrid
4=Amsterdam
5=Lisbon
6=Budapest

Populist Attitudes Intro
19.1. Please indicate for each of the statements below, whether you agree or not:

(1 = Strongly disagree, 2 = Disagree, 3 = Somewhat disagree, 4 = Neither agree nor disagree, 5 = Somewhat agree, 6 = Agree, 7 = Strongly agree)

People-Centrism
20.1. Politicians should always listen closely to the problems of the people.
20.2. Politicians have to spend time among ordinary people to do a good job.
20.3. The will of the people should be the highest principle of a country’s politics.

Anti-Elitism
21.1. The government is pretty much run by a few "big interests" looking out for themselves.
*21.2. Government officials use their power to try to improve people’s lives.

Manichaean Outlook
22.1. You can tell if a person is good or bad if you know their political views.
22.2. The people I disagree with politically are just misinformed.

Nativism (1 = Strongly disagree, 2 = Disagree, 3 = Somewhat disagree, 4 = Neither agree nor disagree, 5 = Somewhat agree, 6 = Agree, 7 = Strongly agree)

23.1. The political and economic elites have failed to protect our cultural identity.
23.2. People who are born in our country should be given priority over immigrants in the employment and housing market.
23.3. People who have immigrated to our country should adjust to our habits, values and traditions here and give up their own culture.

Conspiracy Mentality (1 = Strongly disagree, 2 = Disagree, 3 = Somewhat disagree, 4 =

Neither agree nor disagree, 5 = Somewhat agree, 6 = Agree, 7 = Strongly agree)

24.1. I think that the public is never informed about many of the very important things happening in the world.
24.2. I think that politicians don't usually reveal us the true motives behind their decisions.
24.3. I think that government agencies closely monitor all citizens.
24.4. I think that events, which superficially seem to lack a connection, are often the result of secret activities.
24.5. I think there are secret organizations that greatly influence political decisions.
24.6. Jews or Zionists have engineered the coronavirus as a biological weapon, in order to dominate the world.

Religion vs. Spirituality (1 = not at all, 10 = extremely)
25.1. How religious do you consider yourself?
25.2. How spiritual do you consider yourself?

Appraisals Intro
26.1. The next set of questions is about how you judge the situation concerning the coronavirus crisis and the actions of the government of your country during the past weeks. Please indicate to what extent you agree with the items.

Anger at the Government (1 = Strongly disagree, 2 = Disagree, 3 = Somewhat disagree, 4 = Neither agree nor disagree, 5 = Somewhat agree, 6 = Agree, 7 = Strongly agree)

27.1. I think that our government can be blamed for not reacting fast enough to the outbreak of the coronavirus.
*27.2. I think our government could not have foreseen how fast the coronavirus spreads.
27.3. I am angry at our government for not having taken more far reaching measures to contain the coronavirus.
27.4. I think the measures taken by the government to drastically reduce social contact are inadequate.
27.5. I do not trust my government in the way they handle the coronavirus crisis.
*27.6. I think the government knows what they are doing to fight the coronavirus.
*27.7. I think the information provided by the government is sufficient.

Anger at Transgressors (1 = Strongly disagree, 2 = Disagree, 3 = Somewhat disagree, 4 = Neither agree nor disagree, 5 = Somewhat agree, 6 = Agree, 7 = Strongly agree)

27.8. I think that the main problem is that some people do not follow the rules.
27.9. I think that people who do not follow the rules of the government during the coronavirus crisis are antisocial.
27.10. I think that people who go out in groups during the coronavirus crisis are not properly integrated in our society.
27.11. I think that the coronavirus crisis has escalated, because people do not follow the rules.

Hygiene Measures (General) Intro
28.1. To what extent do you approve of the following measures?

Hygiene Measures (General) (1 = Strongly disagree, 2 = Disagree, 3 = Somewhat disagree, 4 = Neither agree nor disagree, 5 = Somewhat agree, 6 = Agree, 7 = Strongly agree)

29.1. Hand washing for 20 seconds more than 5 times a day.
29.2. Use of disinfectants to clean your hands when soap and water is not available.
29.3. Staying home when you are sick or when you have a cold.
29.4. Wearing a face mask when leaving your house.
29.5. Not visiting elderly people or people with a weak health condition.
29.6. Keeping 1.5 meters distance when you are outside your home.
29.7. Not leaving the house, except for necessary groceries.
29.8. Not inviting other people to your house.
29.9. Not going to any social gatherings with more than 2 people.

Hygiene Measures (Personal) Intro
30.1. What measures do you take yourself?

Hygiene Measures (Personal)
31.1. Hand washing for 20 seconds more than 5 times a day.
(1 = Never, 2 = Very Rarely, 3 = Rarely, 4 = Sometimes, 5 = Occasionally, 6 = Very Frequently, 7 = Always)

31.2. Use of disinfectants to clean your hands when soap and water is not available.
(1 = Never, 2 = Very Rarely, 3 = Rarely, 4 = Sometimes, 5 = Occasionally, 6 = Very Frequently, 7 = Always)

31.3. Staying home when you are sick or when you have a cold.
(1 = Never, 2 = Very Rarely, 3 = Rarely, 4 = Sometimes, 5 = Occasionally, 6 = Very Frequently, 7 = Always)

31.4. Wearing a face mask when leaving your house.
(1 = Never, 2 = Very Rarely, 3 = Rarely, 4 = Sometimes, 5 = Occasionally, 6 = Very Frequently, 7 = Always)

31.5. Visiting elderly people or people with a weak health condition.
(1 = Never, 2 = Rarely, 3 = Occasionally, 4 = Sometimes, 5 = Frequently, 6 = Often 7 = Very Often)

31.6. Keeping 1.5 meters distance when you are outside your home.
(1 = Never, 2 = Very Rarely, 3 = Rarely, 4 = Sometimes, 5 = Occasionally, 6 = Very Frequently, 7 = Always)

31.7. Leaving the house for necessary groceries.
(1 = Never, 2 = Rarely, 3 = Occasionally, 4 = Sometimes, 5 = Frequently, 6 = Often 7 = Very Often)

31.8. Inviting other people to your house.
(1 = Never, 2 = Rarely, 3 = Occasionally, 4 = Sometimes, 5 = Frequently, 6 = Often 7 = Very Often)

31.9. Going to any social gatherings with more than 2 people.
(1 = Never, 2 = Rarely, 3 = Occasionally, 4 = Sometimes, 5 = Frequently, 6 = Often 7 = Very Often)

Civil/Privacy rights (1 = Not at all, 10 = Extremely)
32.1. To what extent do you think the measures by your government violate your civil rights?
32.2. To what extent do you think your government should be allowed to use your GPS mobile phone data in order to combat the coronavirus crisis?

Prosocial Behavior Intro
33.1. Please indicate to what extent you have helped someone during the coronavirus crisis:

Prosocial Behavior
(1 = never, 2 = rarely, 3 = occasionally 4 = sometimes, 5 = frequently, 6 = often, 7 = very often)
34.1. Have you donated money for medical research/equipment regarding the coronavirus?
34.2. Have you helped any of your elderly neighbors/friends, for example in doing groceries?
34.3. Have you taken part in any organized voluntary activities to help people affected by the coronavirus?
34.4. Have you lent any money to someone in need due to the coronavirus?

Moral Reasoning I Intro
35.1. When you see people, who disobey the social distancing rules during the current coronavirus crisis, to what extent would you judge their behavior as follows:

(Judgment) (1 = Not at all, 2 = Low, 3 = Slightly, 4 = Neutral, 5 = Moderately, 6 = Very, 7 = Extremely)

Moral Reasoning I
36.1. Unethical
36.2. Antisocial
36.3. Indifferent
*36.4. Brave
36.5. Disgusting
36.6. Lack of respect for authorities

Moral Reasoning II Intro
37.1. How would you react towards such people?

(1 = Never, 2 = Very Rarely, 3 = Rarely, 4 = Sometimes, 5 = Occasionally, 6 = Very Frequently, 7, Always)

Moral Reasoning II (Reaction)
*38.1. I would look down on them, and say nothing.
*38.2. I would ignore them.
38.3. I would confront them with the rules.

38.4. I would yell at them.
38.5. I would report this to the police.

Moral Reasoning III Intro
39.1. Please read the following sentences and indicate your agreement or disagreement:

(1 = Strongly disagree, 2 = Disagree, 3 = Somewhat disagree, 4 = Neither agree nor disagree, 5 = Somewhat agree, 6 = Disagree, 7 = Strongly agree)

Moral Reasoning III (Values)
40.1. Compassion for those who are suffering is the most crucial virtue.
40.2. When the government makes laws, the number one principle should be ensuring that everyone is treated fairly.
40.3. I am proud of my country's history.
40.4. Respect for authority is something all children need to learn.
40.5. People should not do things that are disgusting, even if no one is harmed.
40.6. It is better to do good than to do bad.
40.7. One of the worst things a person could do is hurt a defenseless animal.
40.8. Justice is the most important requirement for a society.
40.9. People should be loyal to their family members, even when they have done something wrong.
40.10. Men and women each have different roles to play in society.
40.11. I would call some acts wrong on the grounds that they are unnatural.
